# Seasonal Occurrence of African Swine Fever in Wild Boar and Domestic Pigs in EU Member States

**DOI:** 10.3390/v15091955

**Published:** 2023-09-20

**Authors:** Lisa Rogoll, Ann-Kathrin Güttner, Katja Schulz, Hannes Bergmann, Christoph Staubach, Franz J. Conraths, Carola Sauter-Louis

**Affiliations:** Institute of Epidemiology, Friedrich-Loeffler-Institut, Federal Research Institute for Animal Health, Südufer 10, 17493 Greifswald-Insel Riems, Germany; ann-kathrin.guettner@fli.de (A.-K.G.); katja.schulz@fli.de (K.S.); hannes.bergmann@fli.de (H.B.); christoph.staubach@fli.de (C.S.); franz.conraths@fli.de (F.J.C.); carola.sauter-louis@fli.de (C.S.-L.)

**Keywords:** African swine fever, seasonality, surveillance, risk factor, epidemiology

## Abstract

Since 2007, African swine fever (ASF) has spread widely within Europe and beyond. Most affected countries recorded outbreaks in domestic pigs and cases in wild boar. Outbreak data from 2014 to 2021 were used to investigate the seasonal pattern of ASF in domestic pigs and wild boar across affected member states of the European Union, since knowledge of seasonal patterns may provide the potential to adapt prevention, surveillance and control during times of increased risk. In domestic pigs, a yearly peak was observed in many European countries in summer (predominantly in July and August). In wild boar, the patterns showed more variability. In many countries, there was a seasonal peak of ASF occurrence in winter (predominantly in January and December), with an additional summer peak in the Baltic States (predominantly in July) and a further spring peak in Poland (predominantly in March). The observed seasonal effects may be related to the abundance and population dynamics of wild boar and to seasonality in pig farming. Moreover, ASF occurrence may also be influenced by human activities in both domestic pigs and wild boar.

## 1. Introduction

African swine fever (ASF) is caused by African swine fever virus (ASFV), a large DNA virus from the family Asfarviridae [1]. It can cause haemorrhagic disease in suids and affects both Eurasian wild boar and domestic pigs, where it may lead to high case fatality [2,3,4]. From its original sylvatic cycle in sub-Saharan Africa, which involves warthogs and soft ticks of the genus *Ornithodoros* [5], ASF sporadically spilled over to domestic pigs and was occasionally transmitted to Europe in the 20th century, e.g., through the feeding of kitchen waste from ships or aircraft to domestic pigs. Most of these outbreaks were quickly brought under control, but long epidemic spells occurred after the introduction of ASF to Portugal and Spain [6,7]. Also, on the Italian island of Sardinia, ASF remained endemic since 1978 until very recently in a cycle including both domestic and feral pigs [6,8]. However, ASF was absent from the rest of Europe from 1995 until 2007, when ASFV, genotype II, was introduced into Georgia [6]. From there, ASF rapidly spread throughout several Eastern European countries, affecting Armenia and the Russian Federation later in 2007 and Azerbaijan in 2008 [6]. Ukraine and Belarus reported their first cases of ASF in 2012 and 2013, respectively [9]. During this “new” epidemic, Lithuania became affected as the first member state of the European Union (EU) in January 2014 [10], followed by Poland, Latvia and Estonia later that year [10,11,12]. Ever since, ASF has been spreading throughout Eastern, Central and Southern Europe, affecting, among others, the Czech Republic and Romania in 2017; Belgium, Bulgaria and Hungary in 2018 [13]; and Slovakia in 2019 [14]. Subsequently, the first outbreak of ASF in domestic pigs was reported in Greece, and the first cases in wild boar in Germany were confirmed in 2020 [15,16]. More recently, the Italian mainland was affected both in the wild boar and domestic pig sectors in 2022 [17], and Balkan Island countries were affected in 2023. Until recently, Belgium and the Czech Republic were the only two European countries in which ASF was eliminated in wild boar [18,19]. However, ASF was reintroduced into the Czech Republic in late 2022, presumably through wild boar that immigrated from Poland or Germany.

In many countries (e.g., the Baltic States, Bulgaria, Germany, Hungary and Slovakia), the so-called “wild boar habitat cycle” [20] is the main driver of ASF spread and persistence, leading to large case numbers in wild boar and sporadic outbreaks in domestic pigs. In Romania, a different situation was observed: large numbers of outbreaks in domestic pigs were reported, predominantly but not exclusively affecting farms with low levels of biosecurity [21].

Over the years, the seasonality of ASF occurrence has been analysed in a number of studies with variable results. Seasonal peaks in summer for outbreaks in domestic pigs were reported for different countries, e.g., the Baltic States, Poland and Romania [13,22,23]. Several studies detected seasonal patterns in the occurrence of ASF in wild boar with variable results regarding the respective periods and geographical areas [23,24,25,26].

Thus, the aim of this study was to identify seasonal patterns in EU member states that have reported ASF cases to the Animal Disease Information System (ADIS) for the period from 2014 to 2022. Seasonality was analysed in both domestic pigs and wild boar to elucidate the factors affecting these seasonal effects and to compare the results to those of previous studies.

## 2. Materials and Methods

### 2.1. African Swine Fever Surveillance Data

African swine fever surveillance data were used from the EU Animal Disease Information System (ADIS). This system contains information about each confirmed ASF outbreak in domestic pigs and about confirmed ASF cases in wild boar (regardless of the test method) in the European countries reporting to the system. Yet, it is possible that some countries sometimes report several wild boar cases in one record. Due to this potential inconsistency and lack of background information, our analysis was performed under the assumption that one record represents one wild boar case. For the current analysis, information about the country of origin, the date of confirmation and the subspecies (domestic pig or wild boar) was used. Only EU Member States were included in the analysis, as they are considered to have a consistent and reliable reporting system.

Table 1 shows the number of records and the first date of ASF occurrence per country for domestic pigs and for wild boar. The analysed period reaches from the first occurrence of ASF, genotype II, in wild boar in the EU in Lithuania on 24 January 2014, and in domestic pigs in Latvia on 26 June 2014, until 31 December 2022. Since the epidemiological situation in Sardinia differs from that in other European countries (ASFV of genotype I, endemic situation involving free-ranging domestic pigs and wild boar), data from Sardinia were excluded from further analysis.

### 2.2. Data Analyses

All analyses were conducted using the software R [27] with R Studio 4.0.3 [28] as an interface. For descriptive statistics and data management, the R package “tidyverse” [29] was used. Radar charts were produced for each individual country using the absolute and the relative frequency of notifications per month, using the package “fmsb” [30]. In the radar charts, the month of confirmation of ASF was used for the seasonal categorisation, irrespective of the year of the occurrence of ASF. For the detection of seasonal patterns of ASF occurrence, the confirmation dates of ASF for each wild boar record and each outbreak in domestic pigs were converted into a time series and dissected by using the function “decompose” within the R package “stats” [31]. Therefore, a time series is dissected into its components of an overall trend, seasonal effects and remaining random noise by using moving averages to remove the trend and by calculating seasonal figures via averaging over each time unit and over all periods [32]. The Friedman rank test was used to test for seasonality in the time series using the R package “seastests” [33,34]. In addition to global tests for all domestic pig outbreaks and wild boar cases included in the analysis, tests were performed for each country individually considering only the years from the first ASF occurrence until the last ASF occurrence. *p*-values below 0.05 were considered statistically significant and therefore considered as confirmation of seasonality in the tested time series.

## 3. Results

### 3.1. Domestic Pigs

**Figure 1:**
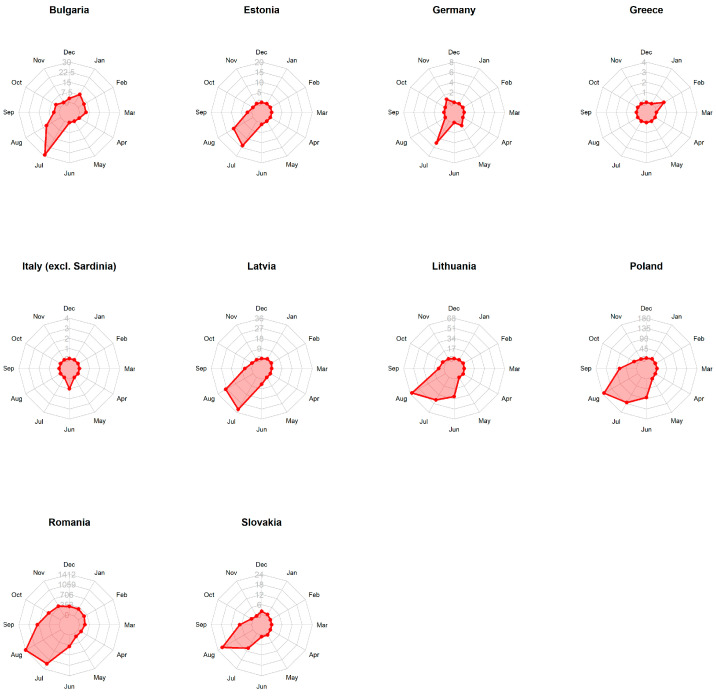
Radar charts of domestic pig outbreaks. Radar charts show the seasonal distribution of number of ASF outbreaks per month in domestic pigs in different European Union countries, irrespective of the year (the scales are adjusted to the maximum number of outbreaks in each country).

In total, the dataset contained 6828 records of ASF outbreaks in domestic pigs. The radar charts (Figure 1) show an increased number of confirmed ASF outbreaks mainly in the summer months (June to September, with the largest numbers of outbreaks in July and August). This pattern was evident for all countries irrespective of the total number of outbreaks per country, except for Greece and Italy. Since only a single ASF outbreak occurred in these two countries (Greece, February 2020; Italy, June 2022) during the study period, seasonality could not be analysed in a meaningful way.

**Figure 2:**
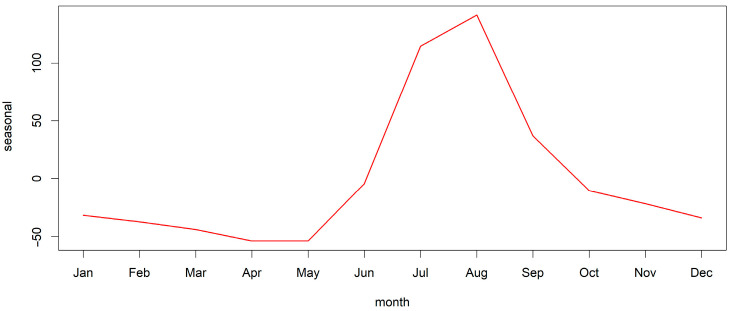
Seasonal pattern of domestic pig outbreaks in the European Union. The figure shows a summary of the seasonal pattern in the monthly course of the year of total ASF outbreaks in domestic pigs in the European Union (except for cases in Sardinia) throughout the time period from the first occurrence in 2014 until 31 December 2022. Further components of the time series are shown in Appendix A, Figure A1.

**Figure 3:**
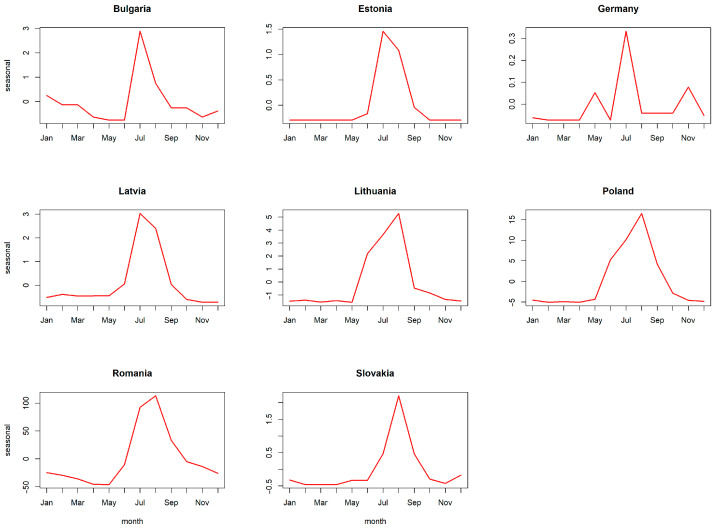
Seasonal pattern of domestic pig outbreaks per country. The figures show a summary of the seasonal pattern in the monthly course of the year of ASF outbreaks in domestic pigs for each country of the European Union, except for Greece and Italy, throughout the time period from the first occurrence in 2014 until 31 December 2022. Since Greece and Italy each had only one outbreak, the detection of a recurring seasonal pattern was not possible.

**Figure A1:**
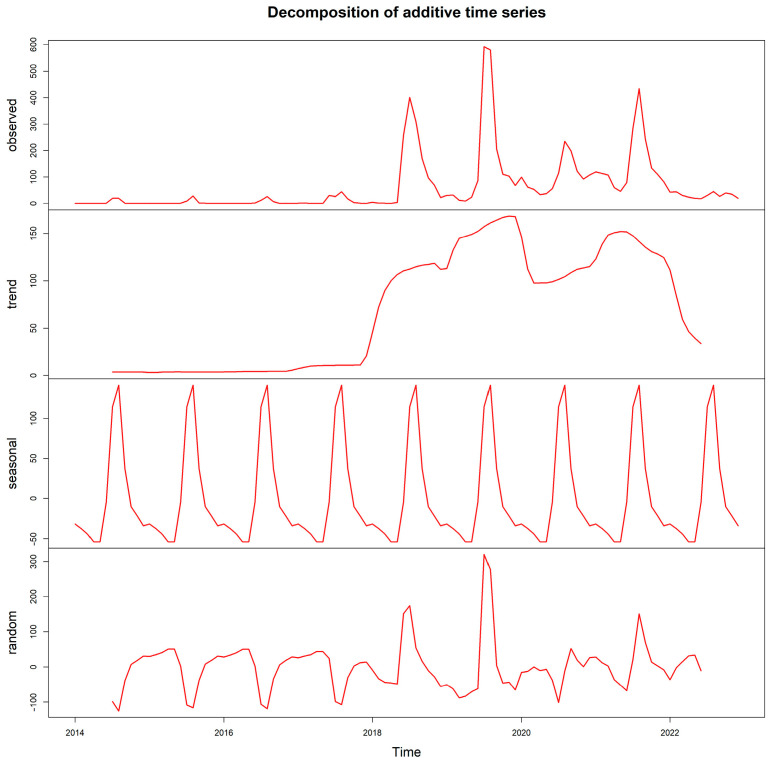
Decomposing the time series of ASF outbreaks in domestic pigs. The figure shows the results for outbreaks in domestic pigs for all countries of the European Union (except for outbreaks in Sardinia) from the first occurrence in 2014 until 31 December 2022 dissected into trends, seasonal patterns and remaining random noise.

Decomposing the time series of all ASF outbreaks in all countries included in the analysis revealed a seasonal pattern with one yearly peak (Figure 2 and Appendix A, Figure A1). Figure 2 shows a summary of the seasonal pattern in the monthly course of the year for all analysed countries during the study period. Appendix A, Figure A1 shows the details of the components of the dissected time series. The seasonality in the time series was confirmed in the global Friedman test (*p* < 0.001) for all countries included in the analysis. The yearly peak according to the dissection of the time series occurred in summer, mainly in July and August, and it could be detected in Bulgaria, Estonia, Germany, Latvia, Lithuania, Poland, Romania and Slovakia (Figure 3). However, the Friedman rank test confirmed seasonality in Latvia (*p* = 0.032), Lithuania (*p* = 0.026), Poland (*p* = 0.004) and Romania (*p* = 0.003) only (Appendix A, Table A1).

### 3.2. Wild Boar

**Figure 4:**
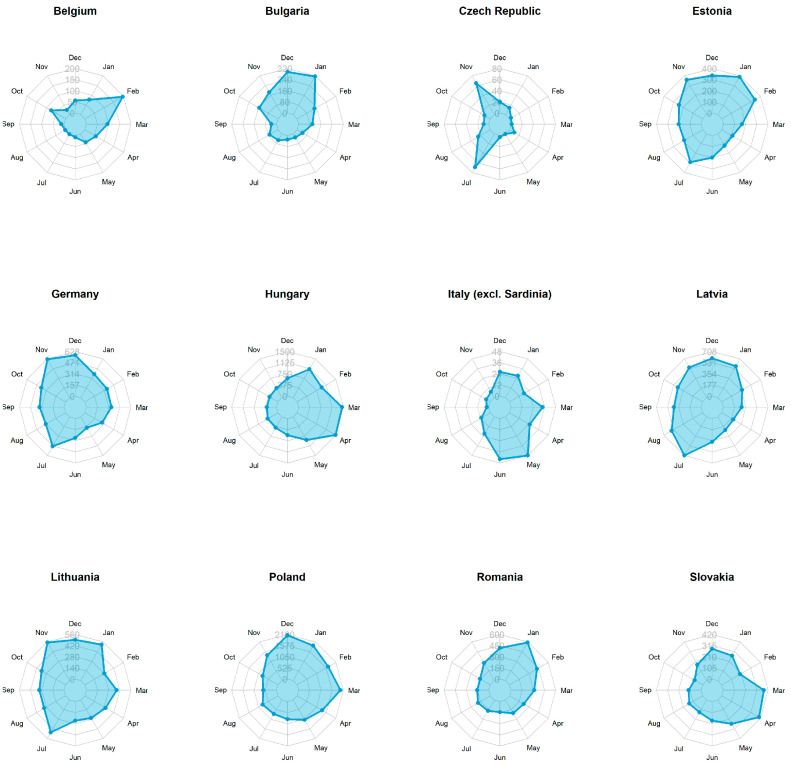
Radar charts of wild boar cases. Seasonal distribution of wild boar tested positively for ASF in different European Union countries across the months (except for cases in Sardinia), irrespective of the year of occurrence (the scales are adjusted to the maximum number of cases in each country).

**Figure 5:**
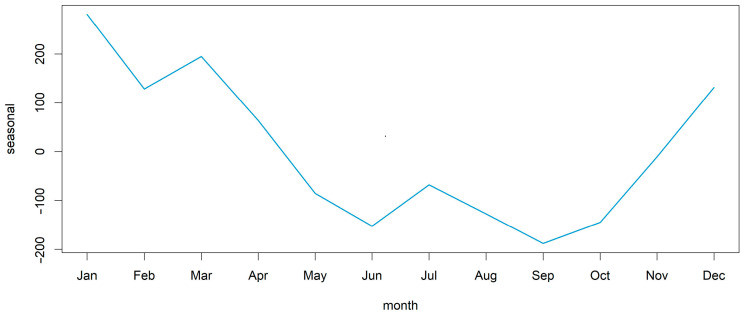
Seasonal pattern of wild boar cases in the European Union. The figure shows a summary of the seasonal pattern in the monthly course of the year of total ASF cases in wild boar in the European Union (except for cases in Sardinia) throughout the time period from the first occurrence in 2014 until 31 December 2022. Further components of the time series are shown in Appendix A, Figure A2.

**Figure A2:**
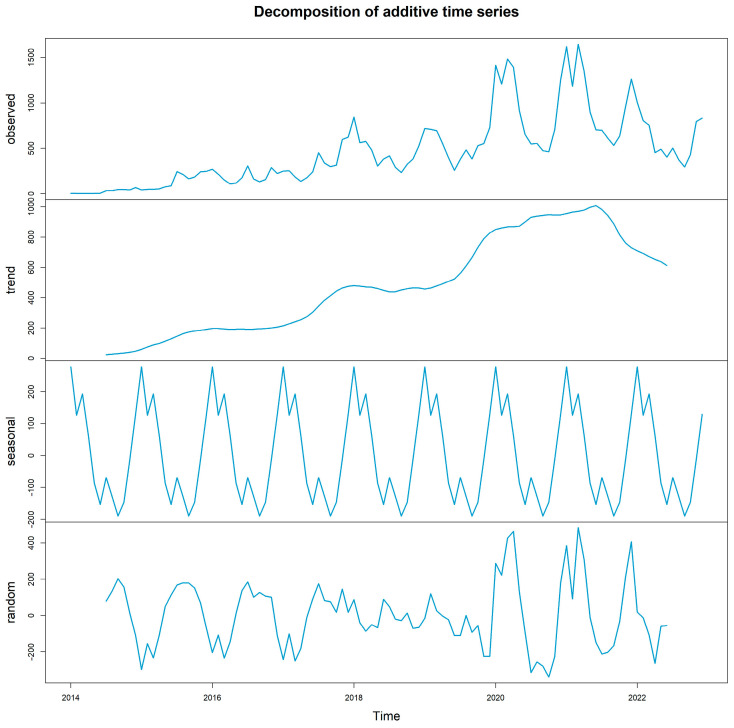
Decomposition of time series of ASF cases in wild boar. The figure shows the results for ASF cases in wild boar in all countries of the European Union (except for cases in Sardinia) from first occurrence in 2014 until 31 December 2022 dissectedinto trends, seasonal patterns and remaining random noise.

The dataset contained a total number of 50,058 records of ASF cases in wild boar. The monthly distribution of the number of ASF cases in wild boar revealed a similar pattern for most of the analysed countries (Figure 4), but the distribution was not as uniform as for the outbreaks in domestic pig holdings. Decomposing the time series of all cases revealed a recurring yearly pattern with one bimodal peak in the winter months and a smaller peak in summer (Figure 5 and Appendix A, Figure A2). The seasonality in the time series was confirmed in the global Friedman rank test (*p* < 0.001) for all countries included in the analysis.

**Figure 6:**
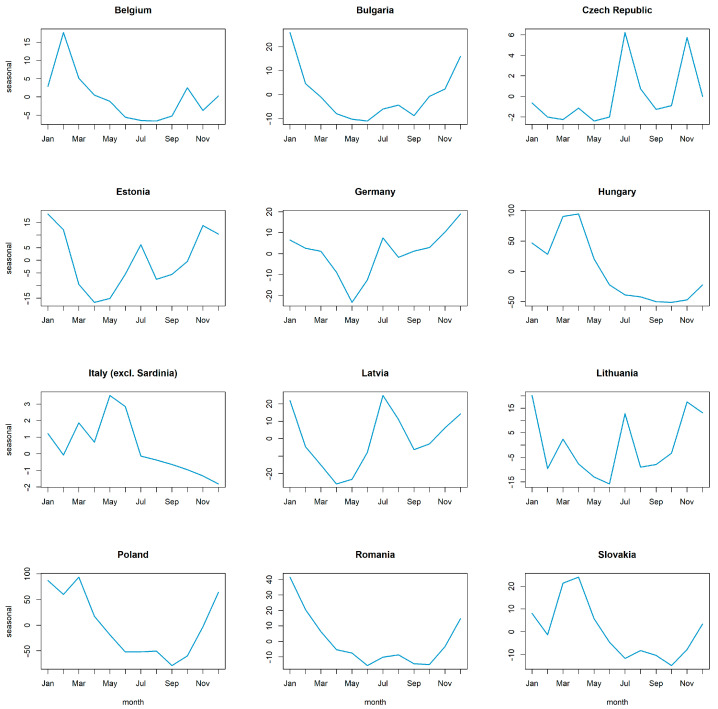
Seasonal pattern of wild boar cases per country. The figures show a summary of the seasonal pattern in the monthly course of the year of ASF cases in wild boar for each country of the European Union (except for cases in Sardinia) throughout the time period from the first occurrence in 2014 until 31 December 2022.

Large case numbers were observed in Bulgaria, Estonia, Germany, Latvia, Lithuania and Romania in the winter months, especially in December and January. Decomposing the data for each country individually (Figure 6) confirmed a seasonal peak in the winter months, mainly in January, for these countries. However, the Friedman rank test confirmed seasonality in Bulgaria (*p* = 0.012), Estonia (*p* < 0.001), Latvia (*p* < 0.001), Lithuania (*p* = 0.018) and Romania (*p* = 0.003) (Appendix A, Table A2).

Germany and the Baltic States (Estonia, Latvia and Lithuania) reported in addition large numbers of ASF cases during summer, mainly in July, leading to an extra summer peak. Only in the Czech Republic was a similar pattern seen. The largest numbers of cases were recorded in July and November, leading to peaks in summer (July) and autumn (November). Belgium recorded most cases in February leading to a winter peak. However, the seasonal pattern in both countries was not significant (Appendix A, Table A2).

By contrast, Hungary and Slovakia detected the largest number of cases in spring, especially in March and April, leading to a peak in this season. Poland reported the largest number of cases from December through to March leading to a winter peak (mainly in January) and a spring peak (mainly in March). The seasonal pattern was significant in Hungary (*p* = 0.009) and Poland (*p* = 0.001) (Appendix A, Table A2). In Italy, most cases occurred in May and June, leading to a seasonal peak in May.

## 4. Discussion

The data used in our study originated from the ADIS database and contained official information about ASF cases in wild boar and outbreaks in domestic pigs in several European countries, particularly in member states of the European Union.

Although the databases hold information on confirmed cases or outbreaks, the test method used to identify and confirm ASF infection is not reported. The detection of ASFV or its genome in samples from domestic pigs or wild boar indicates acute infection at the time of sampling, thus providing relatively precise information on the period when the animal was infected. By contrast, the detection of antibodies only shows that the respective animal had been exposed to ASFV more than at least a few days before sampling. However, animals may remain seropositive for at least several weeks or even months [35,36], so it is not possible to determine the period of infection with ASFV precisely. The inclusion of serological test results for ASFV confirmation may therefore reduce the precision of the information on the true time of infection and may thus hamper seasonality analyses. However, the loss of precision is probably small in the entire dataset, since ASFV is usually detected and confirmed via PCR, i.e., ASFV genome detection. Moreover, it has been shown that the prevalence of ASF-specific antibodies in wild boar is below 2% in the Baltic States in the median [11,23,24,26]. These findings also indicate that the impact of ASFV-seropositive wild boar on the precision of seasonal analyses is very limited in our dataset.

Furthermore, only positive results were reported to ADIS (the case database). For such an analysis, the number of all sampled wild boar and the proportions of positive and negative results would be needed [37]. Moreover, the records in ADIS did not contain background information about the reason for sampling (i.e., passive or active surveillance). Seasonal differences in surveillance intensity may thus influence the overall seasonality patterns observed in our study, but their effect cannot be quantitatively assessed. Furthermore, information about cases and outbreaks were reported to ADIS after the confirmation of ASF, but no information about the putative date of death of the respective animals was included. Especially in wild boar, it cannot be excluded that animals may have died some time before their carcass was detected, which may bias the seasonal pattern. Probst et al. [38] described a method to assess the minimal post-mortem interval, which allows for the approximate time of death of wild boar to be estimated. However, this information was not available in the database. In addition, it is possible that some case records in wild boar each represent more than one ASF-positive wild boar. However, no background information was available, and we consider the likelihood of this issue causing a bias and influencing the seasonal occurrence to be low.

Overall, our analyses showed a relatively uniform seasonal peak of ASF occurrence in wild boar in winter months (mainly in December and January) in the Baltic States, Bulgaria, Germany, Poland and Romania, even though the seasonality in Germany was not significant in the Friedman rank test, presumably due to the shorter time period analysed. This observed seasonality may be an artefact related to an increased surveillance intensity in winter months: Winter is the main season when wild boar are usually hunted. This increases the number of wild boar that can be sampled in this season. Moreover, more hunters dwell in their hunting grounds during this period [39,40], which increases the chance of detecting carcasses of wild boar that succumbed to ASFV infection. In addition, the visibility of wild boar carcasses is higher in winter, since there is less vegetation. Moreover, carcasses decompose slowly because of low temperatures [41]. Altogether, these factors may increase the chance of detecting wild boar carcasses in winter, which could at least in part explain the observed winter peaks of ASF in wild boar.

However, in Lithuania, the ASF prevalence of wild boar found dead in winter was higher than that of animals found in summer between 2014 and 2017 [23] and in 2018 [24]. Also, in Poland, the chance of obtaining ASF-positive test results in wild boar found dead was higher in December and January [25]. This indicates that the detected seasonal pattern of ASF occurrence might not be exclusively caused by the increased sampling of wild boar at this time of the year. The winter peak could also be related to the reproductive behaviour of wild boar and to climate conditions. Since winter is the mating season of wild boar, increased contact rates between animals might lead to a higher risk of ASF transmission. Moreover, carcasses that can pose a risk of infection for living wild boar decompose more slowly in winter than in summer; thus, the capacity for disease transmission through contamination of the environment or interactions of wild boar with carcasses might be increased during this period [41,42,43]. By contrast, the higher temperatures in summer lead to the faster decomposition of carcasses, so they may vanish or be more frequently overlooked, and the low stability of ASFV at temperatures of 20 to 25 °C in different matrices has been shown [44,45,46]. Therefore, ASFV may be preserved for a longer time in winter than in summer months.

Nevertheless, our analyses indicated an additional, dominant summer peak in ASF cases in wild boar in the Baltic States and a less dominant summer peak in Germany, which was not detectable in other countries (except for in the Czech Republic, where the epidemic situation was different). Also, in Poland, we detected a bimodal pattern with peaks in winter (mainly in January) and spring (mainly in March).

Further epidemiological investigations of ASF spread in the Baltic States from 2014 to 2021 showed that the largest numbers of samples from passive surveillance were taken in July in Latvia and Lithuania [26], which might contribute to the increased numbers of positive wild boar in this period. Several studies examined the seasonality in wild boar in Poland with variable results, which could be due to the study areas and periods analysed. Śmietanka et al. [47] observed the largest number of cases and the highest prevalence of ASF in summer and the lowest prevalence in spring and autumn in the period from 2014 to 2015. For the same period, another research group reported the largest number of analysed samples and ASF cases in “July and August but also during February and March” [12]. Similarly, other investigators detected seasonal patterns with peaks in ASF case probability in spring and summer for the period from 2014 to 2016 [48]. By contrast, Frant et al. [25] detected for the years 2017 and 2018 increased chances of obtaining positive results in passive surveillance in winter as compared to summer, while Lu et al. [49] found an increase in the trend for ASF cases in wild boar in October for the period from 2014 to 2017.

Large numbers of samples in summer with large numbers of ASF-positive results could be attributed to increased outdoor leisure activities during summer with increased chances of detecting wild boar carcasses [26]. Yet, the summer peak may also be related to the population dynamics of wild boar: Since spring is the farrowing season, the absolute number of young wild boar is increased during summer. Young pigs are more connected within the population; they have more contacts with wild boar outside their own social group than adults [50] and might have an increased interest in carcasses [51]. Furthermore, the study by Probst et al. [42] showed that most interactions of wild boar with carcasses of their conspecifics were observed in summer and early autumn. Wild boar showed special interest in the insects and maggots present in decomposing carcasses, which are present mostly in the warmer periods of the year [41]. Both factors might increase the risk of disease transmission among young wild boar in summer months.

The patterns of ASF seasonality in wild boar in Belgium and the Czech Republic cannot be compared to the ongoing epidemiological situation in other countries, since the epidemics in these countries were sparked by point infections [52]. The Czech Republic was only affected by ASF for 10 months from 26 June 2017 to 18 April 2018 in a small area of 89 km^2^. During this time, an intensive carcass search and depopulation was conducted, especially in the beginning of the outbreak, as well as in March/April 2018 [18,53]. Similarly, Belgium was affected by ASF only in an area of 620 km^2^ from 13 September 2018 until the last fresh wild boar carcass confirmed positive was found on 11 August 2019. During the outbreak, intensively organised carcass searches and intensified hunting were conducted in the infected zone [19]. Likewise, by the end of our study period, the mainland of Italy had only been affected by ASF in wild boar for one year. The first case was confirmed in January 2022, followed by increased surveillance activities and sampling efforts in the following months [17]. As more data become available, the seasonal patterns of the ongoing epidemics in the Czech Republic and Italy will have to be re-evaluated.

In contrast to wild boar ASF cases, the seasonal pattern for domestic pig outbreaks was relatively uniform in our study, with a summer peak (mainly in July) detected in the Baltic States, Bulgaria, Germany, Poland, Romania and Slovakia, irrespective of the absolute numbers of outbreaks in these countries. However, the seasonality in Bulgaria, Estonia, Germany and Slovakia was not significant in the Friedman rank test, presumably due to comparatively low case numbers in these countries. The summer peak in ASF outbreaks in domestic pigs has also been described in several other studies in EU countries, mainly the Baltic States, Poland and Romania [13,22,23,49,54]. Interestingly, similar summer peaks have also been reported from the Russian Federation [55,56,57] and Sardinia [58].

As for the Baltic States, several studies concluded that mostly domestic pig farms with low levels of biosecurity located in areas where ASF was also present in the wild boar population experienced large numbers of outbreaks in summer months [11,23,54]. In Lithuania and Latvia, mostly backyard pig holdings were affected, whereby shortcomings in biosecurity and the feeding of potentially contaminated fresh grass or crops (which are mainly available in summer) to pigs were considered the main factors for ASFV introduction [11,23]. In Estonia, the number of outbreaks in commercial pig farms exceeded the number of outbreaks in backyard farms, whereby ASFV was most likely introduced by contaminated fomites, such as clothing, vehicles, feed or bedding material [54].

Similar observations were made in domestic pig farms in Poland in the period from 2014 to 2021. The majority of outbreaks in domestic pigs occurred close to areas with previous ASF cases in wild boar [59]. In addition to spillover from the wild boar population, illegal trade, the burial of pigs from non-confirmed outbreaks and the introduction of ASFV by seasonal workers from other Eastern European countries were considered as other potential ways of virus introduction [59].

In Romania, the epidemic situation differs from that in other countries, since primarily outbreaks in domestic pig farms have been reported [21]. Nevertheless, mainly backyard holdings or farms with low levels of biosecurity were affected, and, therefore, transmission routes similar to those mentioned above are considered [13,21].

These observations led to the conclusion that the seasonal patterns of ASF occurrence in domestic pigs are closely linked to wild boar disease dynamics—at least in the Baltic States and Poland—with spillovers of the virus eventually occurring in both directions. Nevertheless, ASF has also occurred in domestic pig farms with high biosecurity settings and/or further away from the epidemic front in the wild boar populations and could not always be attributed to human-associated transmission [13]. This and the detected seasonal patterns with increased case numbers in wild boar and in domestic pigs in spring and summer led to the idea of the potential involvement of an arthropod vector in the transmission cycle of ASF in Europe. This was also concluded from epidemiological investigations by the EFSA, in which they found that most outbreak farms in Romania were located near water sources and that ASF spread increased in the time period following the rainy season, which would provide favourable conditions for insect resurgence [13]. However, no evidence has so far been presented demonstrating that an arthropod vector might currently be involved in ASFV transmission on the European continent [60,61,62,63].

It is known that soft ticks of the genus *Ornithodoros* are competent vectors of ASFV in Africa [64]. In Central Europe and the Baltic States, where soft ticks are almost absent, hard ticks have been checked for their potential role in the transmission of ASFV. In two very common hard tick species, *Ixodes ricinus* and *Dermacentor reticulatus*, no virus replication was observed, but still viral DNA could be detected in the ticks after several weeks, indicating that these ticks are very unlikely to be biological vectors but may play a role as potential mechanical vectors [61].

Also, the stable fly *Stomoxys calcitrans* was identified as a potential mechanical vector for ASFV [65]. Moreover, it has been proven that the ingestion of stable flies that previously fed on ASF-infected wild boar leads to the infection of domestic pigs [63]. However, there are no data from the field showing that this plays any epidemiologically relevant role. In a study by Herm et al., various species of blood-feeding arthropods were collected in an Estonian area with a high prevalence of ASF in wild boar in 2017 and tested for ASFV, all with negative results: no ASFV DNA was detected in *Ixodes ricinus* ticks, *Culicoides punctatus* and biting midges of the *C. obsoletus complex*; in *Aedes* spp., *Anopheles* spp. and *Culiseta annulata* mosquitoes; or in *Haematopota pluvialis* tabanids [62].

## 5. Conclusions

In conclusion, our study shows that seasonal patterns of ASF occurrence in domestic pigs and wild boar in EU countries exist. Knowledge of these patterns provides the potential to adapt control and prevention measures during certain times of high risk and could enable targeted surveillance for the detection of disease in previously unaffected areas and the detection of spread in affected areas. As for outbreaks in domestic pigs, the pattern was relatively uniform, with peaks in the summer months, mainly in July and August. On the contrary, the seasonal pattern for wild boar was not as uniform: most countries showed a peak in the winter months (mainly in December and January), and some showed additional peaks in spring (mainly in March) or summer (mainly in July). These findings suggest that there is a close link between disease dynamics in domestic pigs and wild boar populations, which is dependent on the survival of the virus in the environment, as well as seasonal changes in pig farming and wild boar population dynamics. However, human activities may strongly influence seasonal patterns of ASF occurrence.

## Figures and Tables

**Table 1:** First date of ASF occurrence and total number (#) of records (as of 31 December 2022) used for the analysis per EU member state for domestic pigs and wild boar.

Country	Domestic Pigs		Wild Boar	
	First Date	# Records	First Date	# Records
Belgium	---	0	13 September 2018	648
Bulgaria	31 August 2018	72	23 October 2018	1453
Czech Republic	---	0	26 June 2017	231
Estonia	21 July 2015	28	8 September 2014	2956
Germany	15 July 2021	7	10 September 2020	4554
Greece	5 February 2020	1	---	0
Hungary	---	0	21 April 2018	8899
Italy (excl. Sardinia)	9 June 2022	1	7 January 2022	268
Latvia	26 June 2014	75	26 June 2014	5367
Lithuania	24 July 2014	157	24 January 2014	4475
Poland	23 July 2014	502	17 February 2014	15,306
Romania	31 July 2017	5941	29 May 2018	3267
Slovakia	24 July 2019	44	8 August 2019	2634

## Data Availability

The underlying data from ADIS are not public and only available to the competent authorities responsible for animal health in countries providing data on outbreaks of selected contagious animal diseases and to the European Commission services. Tabulated and cartographical summaries of current outbreak information collected by ADIS are provided by the EU (https://ec.europa.eu/food/animals/animal-diseases/animal-disease-information-system-adis_en, accessed on 6 June 2023). Summaries of used data can be provided upon reasonable request to the authors.

## Appendix A

**Table A1:** Results of the Friedman rank tests of time series of domestic pig outbreaks for each EU member state included in the analysis. The table shows test statistics, *p*-values and the tested periods. The period tested in each country begins in January of the year of the first ASF outbreak and ends in December of the last year of ASF outbreaks. Since Greece and Italy (excluding Sardinia) were only affected in one year of the study period, testing for seasonality with the Friedman rank test was not possible (n.a.). *p*-values below 0.05 are shown in bold.

Country	Test Statistic	*p*-Value	Year of First Occurrence	Year of Last Occurrence
Bulgaria	10.31	0.503	2018	2022
Estonia	5.51	0.904	2015	2021
Germany	7.77	0.734	2021	2022
Greece	n.a.	n.a.	2020	2020
Italy	n.a.	n.a.	2022	2022
Latvia	21.12	**0.032**	2014	2022
Lithuania	21.84	**0.026**	2014	2022
Poland	27.75	**0.004**	2014	2022
Romania	27.83	**0.003**	2017	2022
Slovakia	11.10	0.435	2019	2022

**Table A2:** Results of the Friedman rank tests of time series of wild boar cases for each EU member state included in the analysis. The table shows test statistics, *p*-values and the tested periods. The period tested in each country begins in January of the year of the first reported ASF case and ends in December of the year of the last reported ASF case. Since Italy (excluding Sardinia) was only affected in one year of the study period, testing for seasonality with the Friedman rank test was not possible (n.a.). *p*-values below 0.05 are shown in bold.

Country	Test Statistic	*p*-Value	Year of First Occurrence	Year of Last Occurrence
Belgium	10.92	0.450	2018	2019
Bulgaria	24.26	**0.012**	2018	2022
Czech Republic	2.55	0.995	2017	2022
Estonia	38.12	**<0.001**	2014	2022
Germany	17.92	0.083	2020	2022
Hungary	24.95	**0.009**	2018	2022
Italy	n.a.	n.a.	2022	2022
Latvia	46.81	**<0.001**	2014	2022
Lithuania	22.87	**0.018**	2014	2022
Poland	30.61	**0.001**	2014	2022
Romania	27.98	**0.003**	2018	2022
Slovakia	12.84	0.304	2019	2022
